# Beneficial Effects of American Ginseng on Epididymal Sperm Analyses in Cyclophosphamide Treated Rats

**Published:** 2012-08-31

**Authors:** Hosseini Akram, Firouz Ghaderi Pakdel, Abbas Ahmadi, Samad Zare

**Affiliations:** 1. Reaserch and Clinical Center for Infertility, Shahid Sadoughi University of Medical Sciences, Yazd, Iran; 2. Department of Biology, Faculty of Science, University of Urmia, Urmia, Iran; 3. Department of Physiology, Faculty of Medical Sciences, University of Urmia, Urmia, Iran; 4. Department of Embryology, Faculty of Veterinary, University of Urmia, Urmia, Iran

**Keywords:** Cyclophosphamide, Ginseng, Sperm Motility, Epididymal Sperm, Male Rat

## Abstract

**Objective:**

This study aims to evaluate the protective effects of American ginseng administered by gastric intubation on sperm vital quality in adult male rats treated with cyclophosphamide (CP).

**Materials and Methods:**

In this experimental study, 28 Adult male Wistar rats were assigned to four groups, seven rats in each. The animals allocated to control, CP treated, Ginseng treated and CP-Ginseng treated groups. Rats were treated with CP (6.1 mg/kg/day, i.p) for 6 weeks. American ginseng was used at a dose of 500 mg/kg/day during treatment. Sperm analysis (motion, count, morphology and viability) were evaluated at the end of the experiments. Sperm motion was assessed by Computer-Assisted Sperm Analysis (CASA). The data were analyzed using GB stat software. Probability values of p<0.05 and p<0.01 were considered significant.

**Results:**

The epididymal sperm counts in the groups that received CP showed significant decreases compared to the control group. Also dead and abnormal sperms significantly increased following CP treatment compared with control. The motility of caudal sperm was reduced significantly with CP treatment. Therefore, according to the results of this study, co-administration of CP and American ginseng can improve these parameters.

**Conclusion:**

American ginseng can prevent the cytotoxic effects of CP on sperm quality factors.

## Introduction

Infertility is a biological inability to conceive . Male infertility may also refer to the state of a man who is unable to have potency for fertilization of ovum. Some biological causes of infertility may be treated with medical interventions (1). Sperm disorders are the main causes of male infertility . Besides sperm count, motility parameters are also important factors for the transport of spermatozoa to the egg and its ability to penetrate the zona pellucid, during fertilization. These factors also determine the function of normal mature spermatozoa (2).

Various factors including drugs, chemotherapy, radiotherapy, and apoptosis can affect normal spermatogenesis as well as sperm function, thereby altering male fertility (3). However, chemotherapy must be used to treat some tumors. Cyclophosphamide (CP) is one of the most effective anti-cancer agents for the treatment of malignant and non-malignant disorders. CP is mainly used alongside other chemotherapy agents in the treatment of lymphomas, some forms of leukemia (4) and solid tumors (5). Reproductive toxicity is the major side effect of CP in human and experimental models (6). Long-term treatment for a variety of cancers with CP leads to gonadal toxicity, which can result in sterility in humans (7). Although the precise mechanism of CP induced reproductive toxicity is unknown, previous studies have indicated that CP indisposes the oxidant-antioxidant balance of tissues and increases the production of intracellular reactive oxygen species (ROS) (8-10). The oxidative stress that causes cellular damage is primarily related to the imbalance between the production of ROS and the decrease of antioxidative mechanisms (11).

A number of studies have been conducted on the use of herbal plants in the treatment of infertility. Seeing as these plants usually have low side effects, their administration could be an appropriate approach. Ginseng is the slow-growing aromatic perennial plants belonging to the Panax genus in the family *Araliaceae*. The genus Panax consists of 12 species, 2 found in eastern North America and 10 in Asia. Among them American ginseng, *Panaxquinquefolium* (a family with approximately 700 species), is one of the most heavily traded medicinal plants in North America, and is one of the 10 most commonly used herbal medicines in the United States (12).

Ginseng is a strong antioxidant which has a wide range of actions such as; antiaging, immunoenhancing, antistress, and anti-cancerous (13, 14). According to the Compendium of MateriaMedica, ginseng is a tonic which can be used for the treatment of some disorders such as eliminating bad breath and reducing anxiety (15). Fu and Ji demonstrated that regular ginseng consumption increases an antioxidant capacity of various tissues (16). The active compounds of ginseng are Ginsenosides and triterpenesaponins. Ginsenoside content can vary widely depending on species, location of growth, and growing time before harvest.To date 40 ginsenosides have been identified (17).

A recent study on human and laboratory animals showed that both Asian and American forms of ginseng enhance libido and copulatory performance. These effects of ginseng may not be due to changes in hormone secretion, but to direct effects of ginseng or its ginsenoside components on the central nervous system and gonadal tissues (18, 19). Ginsenosides can facilitate erectile dysfunction (ED) in males (20). Furthermore, a study has also demonstrated a possible effect of ginsenoside-Rc on the motility of sperm (21). Some studies have been conducted to examine ginseng’s protective effects against the toxic effect of environmental contaminants such as 2, 3, 7, 8-tetrachlorodibenzo-p-dioxin (22, 23). Recent studies suggest several beneficial effects of ginseng against the toxicity of chemotherapeutic drugs such as doxorubicin and busulfan in the animal testis (24, 25). Saponins of ginseng can prevent CP-induced genotoxicity and apoptosis in mouse bone marrow cells and peripheral lymphocyte cells (26). To our knowledge, there is no information regarding the effect of American ginseng on the testicular toxicity of CP in mammals. The objective of this work is to evaluate the beneficial impacts of ginseng extract on sperm dynamic parameters in rats treated with anticancer drug cyclophosphamide.Parameters related to sperm motility in male rats include: Average Path Velocity (VAP, µm/s), Straight-Line Velocity (VSL, µm/s), Curvilinear Velocity (VCL, µm/s), Amplitude of Lateral Head displacement (ALH, µm), Beat Cross-Frequency (BCF, HZ), Mean Angle Degree (MAD °).

## Materials and Methods

### Drugs

CP, Eosin-Y, Nigrosin, Phosphate buffered saline were obtained from Sigma-Aldrich Chemicals (St, USA). American ginseng extract was purchased from Xiamen Golden Sun Pharmaceutical Co., Ltd, China.

### Animals

Wistar rats were obtained from the animal center of the Science Faculty of Urmia University (Urmia, Iran). The rats were maintained under standard light (12 hours light /12 hours dark, light on 7.00) with a standard pellet diet and fed and tap water ad libitum. Experimental animals were handled according to principles outlined in the guide to the care and use of experimental animals prepared by the Urmia Council on Animal Care.

### Treatment

Animal groups and dosages were designed according to previous studies (27, 28). Twenty eight adult male rats were divided randomly into four equal groups. Group 1 was used as the control and received normal saline. In group 2, the rats were intraperitonially administered with CP at a dose of 6.1 mg/kg/day. Group 3 received American ginseng dissolved in normal saline at a dose of 500 mg/kg/day by gastric intubation for a period of 6 weeks. Group 4 rats received American ginseng 1 hour prior to the administration of CP. At the end of the experimental period, all animals were sacrificed by decapitation and their epididymides were removed immediately; the caudal epididyms were used for sperm analysis. Briefly, epididymal sperm were collected by slicing the caudal epididyms in HTF + 4mg/ml BSA, and incubated in a CO_2_ incubator (5% CO_2_ in air at 37℃) for 10 minutes, allowing sperm to swim into the medium. The epididymides were processed for the following analysis.

### Sperm count analysis

To assess content of spermatozoa, the heads of spermatozoa were counted hemocytometrically. 5 µl aliquot of epididymal sperm was diluted with 95 µl of diluent. 10 µl of the diluted sperm suspension was transferred to each counting chamber and was allowed to stand for 5 minutes in a humid chamber to prevent drying. Heads of sperms were counted with a light microscope at ×400 (27).

### Sperm morphological study

A part of sperm suspension was used for preparing smears to evaluate the sperm morphological abnormalities. One drop of sperm suspension was added into an equal volume 1% eosin-y 5% nigrosin, mixed together, smears were prepared on clean glass slides and air-dried. Two hundred sperm cells were examined per animal to determine the morphological abnormalities at ×400 magnification (27, 29). Any disorders in the morphology and structure of either head or tail or both were considered as abnormal.

### Sperm viability assay

In order to study the sperm viability, 20 µl of the sperm suspension was mixed with 0.05% eosin -y. Slides were prepared and incubated for two minutes at room temperature before being evaluated using a light microscope at ×400 magnification. Two hundred sperm were counted for each sample and viability percentages were calculated (27). Dead sperm appeared pink and live sperm were not stained

### Sperm motility

Sperm motion analysis by the Computer-Assisted Sperm Analysis (CASA) system, (Hamilton-Thorne IVOS system) was performed as follows: 10 µl of sperm diluted solution were placed on observation chambers for CASA analysis using the Wilei color analysis software (30-32). For each animal, 4 slides were analyzed. The parameters used for this study were: (VAP, µm/s), (VSL, µm/s), (VCL, µm/s), (ALH, µm), (BCF, HZ), (MAD °).

Results were analyzed by GB-Stat statistical software. Significance of difference between groups was evaluated using ANOVA and Tukey post test. P<0.05 was considered to be statistically significant.

## Results

As shown in table 1, treatment of CP decreased number of sperm, while increasing the number of dead and abnormal sperm compared to the control. Co-treatment of cyclophosphamide and American ginseng improved these alterations.

**Table 1:** The Effects of CP and American ginseng on the sperm status of male rats


	Count sperm (ml/10^6^)	Dead sperm (%)	Abnormal sperm (%)

Con	108.86 ± 19.45	30.92 ± 4.83	25.37 ± 3.90
CP	76.69 ± 13.62^*^	56.49 ± 5.63^**^	54.12 ± 7.83^**^
Gin	117.34 ± 28.95^*^	25.9 ± 5.55	17.58 ± 5.53
CP+Gin	85.33 ± 30.90	28.96 ± 5.59	27.37 ± 3.79


The values are expressed as mean ± SEM. *Significantly different from control groups (p<0.05). ** Significantly different from control groups (p<0.01). Con;Control, CP; Cyclophosphamide, Gin;Ginseng and CP + Gin; Cyclophosphamide + ginseng.

**Table 2:** Effects of CP and American ginseng on the sperm motility parameters of male rats


	VCL (µm/s)	VSL (µm/s)	VAP (µm/s)	BCF (Hz)	MAD (°)	ALH (µm)

Con	263.31 ± 8.47	108.91 ± 11.88	162.14 ± 6.85	13.13 ± 3.68	67.78 ± 11.61	28.37 ± 6.84
CP	238.92 ± 7.2^**^	88.62 ± 7.61^*^	148.72 ± 4.72 ^*^	16.55 ± 3.37	83.31 ± 5.91 ^*^	17.97 ± 2.42 ^*^
Gin	271.95 ± 12.15 ^**^	113.61 ± 12.25 ^*^	172.33 ± 9.20 ^**^	11.52 ± 5.09	60.13 ± 15.59 ^*^	30.84 ± 11.21
CP + Gin	260.14 ± 10.09	94.06 ± 4.13	161.12 ± 13.68	14.53 ± 4.75	67.53 ± 7.20	26.95 ± 7.62


VCL; Track velocity, VSL; Progressive velocity, VAP; Path velocity, BCF; Beat frequency, MAD ; Mean angle degree and ALH ; Lateral amplitude. The values are expressed as mean ± SEM. *Significantly different from control groups, (p<0.05). ** Significantly different from control groups, (p<0.01). Con; Control, CP; Cyclophosphamide, Gin; Ginseng and CP + Gin; Cyclophosphamide + ginseng.

Results of sperm motion analysis are shown in table 2. Treatment of the animals with CP caused a significant decreased in VCL, VSL, VAP, ALH parameters (p<0.05) whereas, MAD was significantly increased (p<0.05). In this study no significant difference was observed in BCF between groups. Our finding indicated that co-treatment American ginseng could restore all changes.

## Discussion

The susceptibility of chemicals drugs and/or compounds to impair reproductive processes in laboratory animals and humans is of great concern to toxicologists and the public. CP is a common chemotherapy agent that is used to treat cancer malignancies and acts as an immunosuppressive agent. CP is mutagenic and induces many side effects such as alteration of male spermatozoa leading to sterility (33). The metabolism of CP produces two active metabolites, phosphoramide mustard and acrolein. Some studies have reported that CP can induce germ cell toxicity by generating ROS (27, 33). ROS induce a significant reduction in semen quality by decreasing sperm count and motility. They can also increase sperm defects and impairment of antioxidant synthesis (11). There is evidence that CP treatment of male animals can affect sperm characteristics as we was reported in our findings (34). Furthermore, we observed a decrease in the six parameters of sperm motility, VSL, VCL, VAP and ALH due to CP treatment. A study by Mohammad Gholizad has confirmed that these parameters changed after chemotherapy. CP caused an increase in the number of dead and amorphous sperm and decreased sperm count. These findings indicate that CP induced cytotoxicity in sperm cells and this is in agreement with other reports (34).

CP can induce the formation of abnormal sperm cells; the increase in sperm abnormalities indicates that CP induced DNA damage in germ cells leading to altered sperm morphology. Sikka has reported that peroxidation of critical thiol groups in protein can alter the structure and function of spermatozoa (35). The decrease in sperm count is an important factor leading to male infertility (36).

Selvakumar and colleagues reported that the decrease in sperm counts is due to the generation of ROS by CP and the consequential elimination of sperm cells at different stages of development (37). Endogenous and exogenous antioxidants may protect cells and tissues from destructive effects of ROS and other free radicals. Previous studies reported that sperm disorders can be improved by exogenous antioxidants/ROS scavengers (38). Antioxidant properties of American ginseng are well known (39). American ginseng or its extracts have been reported to exhibit free radical scavenging activities and can prevent lipid peroxidation (40).

The results of this current study also suggest that the testicular toxicity induced by CP was significantly recovered by American ginseng. Zhang et al. reported ginsenosides increases sperm motility in fertile and asthenozoospermic human (41). Number of motile spermatozoa and their speed directly correlates to fertilization success (42). Kato et al. reported that the velocity parameters (VSL, VCL and VAP) directly express sperm motion (43) and decline in sperm velocity, percentage of motile sperm, BCF and ALH parameters can also adversely affect fertility (44). This may explain why fertility remains a problem even in patients who are not oligospermic (45). Generation of ROS by CP decomposes sperm plasma membrane and is therefore responsible for loss of sperm motility (46), which is presumably caused by a rapid loss of intracellular ATP leading to damage in sperm flagellum. Activity of Na^+^-K^+^-ATPase is highly sensitive to ROS, thus depletion of Na^+^-K^+^-ATPase can be a good reason for the reduction of sperm motility. The existence of morphologic abnormalities and decreased sperm viability has been associated with ROS production (47, 48). Also defects in the flagella, changes in motility and morphology of spermatozoa, are likely associated with infertility. Sperm cells are more susceptible to peroxidative damage because of high concentration of polyunsaturated fatty acids and low antioxidant capacity (49). Membrane-associated polyunsaturated fatty acids such as sperm are readily attached by ROS. Peroxidation of membrane lipids can disrupt membrane fluidity and cell compartmentation, which can result in cell lysis. Reduction in sperm counts, the existence of morphologic abnormalities could be due to the generation of ROS by CP (48, 50).

These results have clearly shown that CP treatment has a deleterious impact on the quality of spermatozoa in the male rat, resulting in a decrease in sperm numbers and motility and an increase in the number of morphologically abnormal sperm cells. American ginseng treatment exhibits therapeutic effects on sperm parameters in rats treated with CP. It seems that American ginseng acts as a free radical scavenger and therefore causes these alterations (51).

## Conclusion

The results of our work suggest that American ginseng acts as a potent antioxidant in the protection of rat sperm cells against oxidative stress induced by cyclophosphamide. Furthermore, it protected the animals from the adverse consequences of CP exposure based on parameters of testicular toxicity.
